# Strategies for the Emergency Treatment of Pregnant Women with Neurological Symptoms during the COVID-19 Pandemic

**DOI:** 10.14336/AD.2022.0718

**Published:** 2023-04-01

**Authors:** Haojun Yang, Yishu Fan, Ziqing Zhu, Haiyue Wu, Zhuohui Chen, Xinhang Hu, Tong Wu, Mengqi Zhang

**Affiliations:** ^1^Department of Neurology, Xiangya Hospital of Central South University, Changsha, Hunan, China; ^2^National Clinical Research Center for Geriatric Disorders, Xiangya Hospital, Central South University, Changsha, China

**Keywords:** Corona Virus Disease 2019, epidemic prevention and control, pregnant women, CNS disease

## Abstract

Coronavirus disease-19 (COVID-19) has been spreading all over the world for more than two years. Though several kinds of vaccines are currently available, emergence of new variants, spike mutations and immune escape have raised new challenges. Pregnant women are vulnerable to respiratory infections due to their altered immune defence and surveillance functions. Besides, whether pregnant persons should receive a COVID-19 vaccine is still under debate because limited data are available on the efficacy and safety of receiving a vaccine during pregnancy. Physiological features and lack of effective protection making pregnant women at high risk of getting infected. Another concern is that pregnancy may trigger the onset of underlying existing neurological disease, which is highly similar to those neurological symptoms of pregnant women caused by COVID-19. These similarities interfere with diagnosis and delay timely and effective management. Therefore, providing efficient emergency support for pregnant women suffering from neurological symptoms caused by COVID-19 remains a challenge among neurologists and obstetricians. To improve the diagnosis and treatment efficiency of pregnant women with neurological symptoms, we propose an emergency management framework based on the clinicians’ experience and available resources. This emergency care system aimed at addressing the conundrums faced by the emergency guarantee system under COVID-19 pandemic and could serve as a potential multisystem project for clinical practice and medical education.

## 1. Introduction

Since the identification of the first coronavirus disease (COVID-19) case in Wuhan, Hubei Province, China, in December 2019, this life-threatening coronavirus, acute respiratory syndrome coronavirus (SARS-CoV), has caused over 207 million infections and more than 4 million deaths [1]. To prevent the infection, many countries have imposed strict non-essential movements restrictions and lockdowns, which cause severe economic consequences [2]. Currently, 33 vaccines based on different mechanisms have already been approved and applied in different countries [3], which to some extent help to prevent the spread of this disease. However, the emergence of new variants, spike mutations and immune escape of SARS-CoV have raised new challenges [4, 5]. For example, the Delta variant (B.1.617.2 lineage) and the Omicron variant (B.1.1.529 lineage) put new threats and re-increase the infected numbers [6]. Considering this, Covid-19 probably remains a life-threatening disease in the next few years, which deserve our attention and efforts.

Pregnant women are a special group of people who have higher susceptibility to SARS-CoV as well as poor prognosis and higher mortality rates due to their immunocompromised status and pathophysiological alterations [7]. In Washington State, the infection rate in pregnant people was even 70% higher than nonpregnant women of similar age [8]. According to data from the United States Centers for Disease Control and Prevention COVID-19 surveillance system, compared with nonpregnant women of reproductive age, pregnant women infected with COVID-19 were significantly more likely to be admitted to an intensive care unit (ICU) (10.5 versus 3.9 per 1,000 cases), receive invasive ventilation (2.9 versus 1.1 per 1,000 cases), receive extracorporeal membrane oxygenation (ECMO) (0.7 versus 0.3 per 1,000 cases), and die (1.5 versus 1.2 per 1,000 cases) [9]. In the period of Delta variant predominance, the risk of ICU admission, invasive ventilation, ECMO and risk of death were even higher compared with the pre-Delta period among pregnant women [10]. Therefore, ensuring their safety and guaranteeing a safe process of pregnancy, delivery, and puerperium are of great importance for maternal and newborn’s health. On 8 February 2020, the Chinese National Health Commission released the ‘Notice on Strengthening the Treatment of Maternal Illness and Safe Delivery under COVID-19 Pandemic’, emphasising the importance of strengthening prenatal health care in pregnant women during the pandemic [11].

Neurological complications in patients with COVID-19 includes headache, dizziness, impaired consciousness, acute cerebrovascular disease, epilepsy, hyposmia/anosmia, hypogeusia/ageusia, myalgia, and Guillain-Barre syndrome [12]. Pregnant women also face the risk of developing neurological symptoms [13]. However, pregnant women also have risk of developing neurological complications during pregnancy and puerperium which is unrelative to COVID-19 infection [14, 15]. Considering that the treatment for these two situations is quite different, it is critical to figure out whether neurological symptoms are caused by virus infection or changes during pregnancy itself. This efficient differentiation will help to improve the pregnancy outcomes as well as reduce the exposure risks and nosocomial infection rates. Herein, we propose an emergency management framework for pregnant women with neurological symptoms to improve the diagnosis and treatment efficiency. This emergency management system could also serve as a potential multisystem project for clinical practice and medical education.

## 2. Effects of the COVID-19 pandemic on pregnant women

### 2.1 Physiological characters of pregnant women

Pregnancy brings significant anatomical and physiological changes to the mother’s body to accommodate and provide adequate blood, nutrition, and oxygen to the developing foetus. These changes involve multiple organs and multiple systems and affect pharmacokinetics and pharmacodynamics of drugs [16]. General changes include increased plasma volume, decreased platelet count, increased coagulation tendency, increased cardiac output, marked fall in systemic vascular resistance (SVR), increased glomerular filtration rate (GFR), altered endocrine spectrum and metabolism of nutrients [17-19]. Importantly, because the fetus is semi-allogeneic, the maternal immune system undergoes profound changes during pregnancy to avoid compromising fetal and maternal health [20]. Pregnant women’s immune responses changes to favor an anti-inflammatory response, featured by decreased activities of T-cell and natural killer cells, increased number of the regulatory T cells and increased levels of estrogens and progesterone [21]. Elevated progesterone levels is also thought to cause decreased sphincter tone in the lower esophagus, which put pregnant women at an increased risk of aspiration pneumonia [22]. All these changes, together with enhanced drug elimination and decreased exposure to total drugs (bound and unbound to plasma proteins) at a given dose during pregnancy, significantly increase maternal morbidity from respiratory diseases [23]. In addition, as has been well known that increased maternal age is associated with higher risk of infertility, pregnancy complications, spontaneous abortion, congenital anomalies, perinatal complications and adverse pregnancy outcomes [24]. With the launch of the two- and three-child policy in China, advanced maternal age is becoming an increasingly relevant issue, adding more difficulty to clinical prenatal care especially under COVID-19 pandemic.

### 2.2. Clinical symptoms of pregnant women with COVID-19

The major clinical symptoms of COVID-19 include fever, fatigue, and dry cough [25]. However, some patients present non-respiratory symptoms, such as neurological, gastrointestinal, and cardiovascular manifestations [26-28]. Information from the National Health Commission of China showed that the most common symptoms in pregnant women with COVID-19 were fever (75%) and cough (73%). Other symptoms include chest tightness (18%), fatigue (17%), dyspnea (7%), diarrhea (7%) and headache (6%) [29]. Symptoms of non-pregnant adult patients were fever (98%), cough (76%), dyspnea (55%), fatigue (44%), headache (8%), and diarrheal (3%) [30]. Neurological symptoms manifestations, and complications such as headache, seizures, altered mental status, stroke, paralysis and loss of consciousness could be observed in some cases [31], while acute cerebrovascular disease, loss of consciousness, and muscle damage were found in patients with a severe infection [32-34]. The actual cause of neurological symptoms in COVID-19 patients remained unknown. Although hypoxemia caused by pneumonia can similarly cause the aforementioned neurological symptoms, the neuro-invasive potential of SARS-CoV-2 may be the reason of acute respiratory failure in COVID-19 patients [33].

## 3. Different neurological symptoms presented in pregnant women with and without COVID-19

Pregnancy is a unique physiological process for women. As we mentioned above, pregnant women undergo changes involve in multiple organs and multiple systems. They experience pathophysiological changes in their coagulation system along with pregnancy-specific organ damage, thus causing neurological symptoms. As has been demonstrated, SARS-CoV-2 utilize the angiotensin-converting enzyme 2 (ACE2) as an entry point to the targeted cells [35]. Human neurons were found to express ACE2 on the surface, which indicated the neuro-invasive potential of SARS-CoV-2 [36]. The neurological symptoms in COVID-19 patients may be caused by direct viral neurological injury or indirect neuroinflammatory and autoimmune mechanisms [37]. Thus, similar neurological symptoms could be observed in pregnant women with and without COVID-19. Since the treatments towards these two situations are quite different, more attention should be paid to carefully distinguishing the cause of those symptoms. Common neurological disorders during pregnancy are summarised as follows to provide a basis for early diagnosis and treatment in patients.

### 3.1 Cerebral venous sinus thrombosis

Cerebral venous sinus thrombosis (CVST) is thrombosis of cerebral venous which results in cerebrospinal fluid (CSF) absorption impairment and elevated intracranial pressure, which leads to cerebral oedema, infarction and haemorrhage [38]. CVST is one of the severe complications during pregnancy, threatening the lives of women and foetuses [39]. Typical symptoms include headache, altered consciousness, vomiting, seizures, cranial nerve involvement, aphasia, paresis, and paresthesias [40, 41]. The possibility of CVST should be considered if the symptoms are observed in pregnant women. Some red flags to identify pregnant women presenting CVST have been suggested in the diagnostic algorithm for pregnant and postpartum patients with acute neurological symptoms [42]. Recently, more cases of COVID-19-associated CVST have been showing up, indicating CVST as a potential thromboembolic complication of COVID-19 [43, 44]. It is worth noting that the COVID-19 infection preceded the symptoms of CVST by nearly two weeks, which indicates that the hypercoagulable effect of COVID-19 may be related to the initial infectious event [45]. SARS-CoV-2 infection was considered as a potential risk factor of CVST. Elevated D-dimer levels and platelet dysfunction with potential hyperactivation via inflammatory cascades were observed in patients infected by SARS-CoV-2 [46-48]. These changes put patients at greater risk. Given that the therapeutic methods of CVST differs according to its pathogenesis, it is necessary to distinguish pregnant women diagnosed with CVST from those once infected with COVID-19.

### 3.2 Epilepsy

Epilepsy is a brain condition characterized by the recurrence of unprovoked seizures. It is caused by repeated, excessive, and aberrant synchronous discharge by neurons [49]. Seizures during pregnancy complicate <1% of all gestations but they are associated with severe outcomes [50]. 2%-4% epileptic pregnant women will experience tonic-clonic seizures during or within 24 hours after delivery, often resulting in maternal death [51]. If epileptic seizures occur during pregnancy, pregnancy should be terminated. Under COVID-19 pandemic, de novo seizures were found to occur in people infected with SARS-CoV-2 in a variety of forms [52]. Potential mechanism for this complaints is that pro-inflammatory cytokines caused by SARS-CoV-2 infection could reduce GABA levels, impairs the function of ion channels, and induce aberrant synchronous discharge by neurons [53]. Therapies should focus in eliminating pro-inflammatory cytokines and protect neuron from being damaged. This is quite different from those epileptic pregnant without virus infection.

### 3.3 Autoimmune encephalitis (AE)

AE is a group of inflammatory brain diseases s characterised by limbic and extra-limbic dysfunctional symptoms, which is caused by autoantibodies against synaptic receptors, iron channels or surface proteins of neurons [54]. The clinical manifestations of AE include dyskinesias, autonomic disturbance, central hypo-ventilation psychiatric disorders, epileptic seizures, and loss of consciousness. Diagnosis of AE is challenging because the clinical presentations of AE always overlap with other neurological diseases. Besides, the clinical manifestation of AE is complexity and keep changing [55]. Pregnancy changes the maternal immune system to an anti-inflammatory and autoimmune suppressive state. Besides, Dariush et. al. found that pregnancy level of estrogen attenuates experimental via upregulating the expansion of Treg and Th2 cells [56]. Therefore, AE is rare during pregnancy, and good results can be achieved through immune therapies [57, 58]. When combined with COVID-19 infection, things are quite different. The neuro-invasive potential of SARS-CoV-2 makes it possible to induce various spectrum of autoimmune encephalitis. A prospective study showed that AE related symptoms were observed a few days or weeks after the end of the viral infection [59]. When a pregnant women come with the clinical presentations of altered mental status, impaired consciousness seizure, motor, and reflex abnormalities, attention should be paid to distinguishing autoimmune diseases and SARS-CoV-2 caused neuroinflammation.

### 3.4 Central nervous system (CNS) infection

CNS infection is common in pregnant women due to their altered immune system function. It is caused by a broad-spectrum of pathogens such as bacteria, viruses, fungi, parasitic worms like toxoplasma, mycoplasma, chlamydia, moulds, and rickettsia [60, 61]. Clinical symptoms of these infections include chills, fever, upper respiratory tract infection symptoms and CNS system related symptoms such as headache, vomiting, convulsion, loss of consciousness, positive meningeal irritation signs and convulsions [62]. Severe neurological sequelae without timely treatment often threaten the safety of the foetus. Pregnant women present with CNS symptoms should be carefully examined to exclude neurological symptoms caused by COVID-19 infection. Patients’ epidemiological history inquiry, physical examination, complete blood count, routine cerebrospinal fluid test, biochemical test, pathogenic microorganisms, and imaging tests are useful clinical methods. Imaging examinations such as CT and MRI should be applied if necessary. And for suspected COVID-19 patients, qRT-PCR testing of cerebrospinal fluid should be carried out as soon as possible. Next-generation sequencing and cerebrospinal fluid culture can be used for etiological detection [63].

## 4. Treatment of pregnant women with CNS symptoms

Multidisciplinary collaboration should be applied to pregnant women with CNS symptom. If headache, delirium, and epileptic seizures occur, dehydration, neuroprotective, anticonvulsant, and antipsychotic treatments should be added [63]. Generally, clinical management of pregnant women with COVID-19 is similar to that of nonpregnant women. On the basis of supportive treatment, such as bed rest, nutrition supply and close monitoring of vital signs, antiviral treatments should be applied to those suspected or confirmed with virus infection. Commonly used antiviral drugs include interferon-α, levetiracetam, lamotrigine, atazanavir, ritonavir, ribavirin, raltegravir, acyclovir, tenofovir, valganciclovir and valacyclovir [64]. The indicated use and pregnancy safety data of common applied medications can be found in published studies [65, 66]. Based on clinical evidence, levetiracetam and lamotrigine are the safest antiviral drugs during pregnancy [67, 68]. Compassionate-use of remdesivir in pregnant and postpartum women with severe COVID-19 brought high recovery rates with a low rate of severe side effects [69].α-interferon was found to inhibit the developmental potential of placental trophoblast stem cells [70]. If used in the first trimester, it may hinder fetal growth and development. Ribavirin may damage human red blood cells and cause hemolysis and may have teratogenic effects, which should be avoided in the first 6 months of pregnancy [71]. Short-term, low-dose glucocorticoids can be used, but an appropriate dose is necessary because excessive use is harmful to maternal and foetal health [72]. However, when it comes to COVID-19 infection, pregnancy or lactation shouldn’t be an indication for limited drug use. To prevent severe outcomes, effective antiviral therapy such as remdesivir should not be withheld even with limited albeit reassuring safety data [1]. In addition, several types of monoclonal antibodies are available for COVID-19 treatment which can also be applied for pregnant women [73]. Prophylactic anticoagulation is recommended for hospitalized pregnant patients [74]. For pregnant women infected with COVID-19 who present a fever, acetaminophen should be used with caution. It is found to cause attention deficit hyperactivity disorder and neurodevelopmental disorders in offspring [75, 76]. Recent human studies confirmed that the administration of ACEIs or ARBs did not increase the expression of ACE2 [77-79]. Therefore, ACEIs and ARBs can be used as anti-hypertensive medications in pregnant women with hypertension despite COVID-19 infection. Does and types of these pharmacological treatment in pregnant women with COVID-19 are not specific [80]. Other drugs that do not pose a theoretical risk of promoting COVID-19 proliferation appear to be a reasonable strategy to optimize patient outcomes, such as verapamil [81].

Despite the similarities, there are some important differences in the treatment of pregnant and nonpregnant persons. For example, pregnant women require a higher oxygen saturation, generally should be maintained at 95% or greater on room air. In addition, some COVID patients who receive mechanical ventilated benefit from prone positioning. Considering the body shape changes of pregnant women, some possible modifications such as positioning in the left lateral decubitus position could be more benefit and safer. For additional information, please see the www.covid19treatmentguidelines.nih.gov/.

Pregnant women with high-risk conditions are recommended to receive additional necessary prenatal care and antenatal surveillance. However, continuous fetal monitoring should be considered only after fetal viability, when delivery would not compromise maternal health, or as another noninvasive measure of maternal status. Clinical guidelines proposed by the National Institutes of Health and the Society for Maternal and Fetal Medicine (SMFM) emphasized that COVID is not an indication for delivery and should neither alter the timing nor the mode of delivery. But the delayed delivery may be considered until the mother tests negative for COVID to decrease the transmission. More clinical guidelines could be refered at www.smfm.org/covidclinical.

## 5. A framework to improve emergency managements for pregnant women with neurological symptoms during COVID-19 epidemic

Pregnant women are extremely susceptible to various respiratory pathogens and linked to a higher incidence of severe Covid-19 complications. Besides, the exclusion of pregnant women from the COVID vaccine trials has brought the worry of vaccine safety and efficacy [82]. Many pregnant women are hesitant to be vaccinated because of safety concerns, making them one of the high-risk populations that are susceptible to COVID-19. Neurological symptoms are common complications during pregnancy which are similar to those caused by COVID-19 infection such as fever, headache, convulsions, and loss of consciousness. The misdiagnosis may lead to delayed treatment and increased risk of exposure. To solve this problem, we propose a framework to improve emergency managements for pregnant women with neurological symptoms during COVID-19 epidemic.

**Figure 1.**
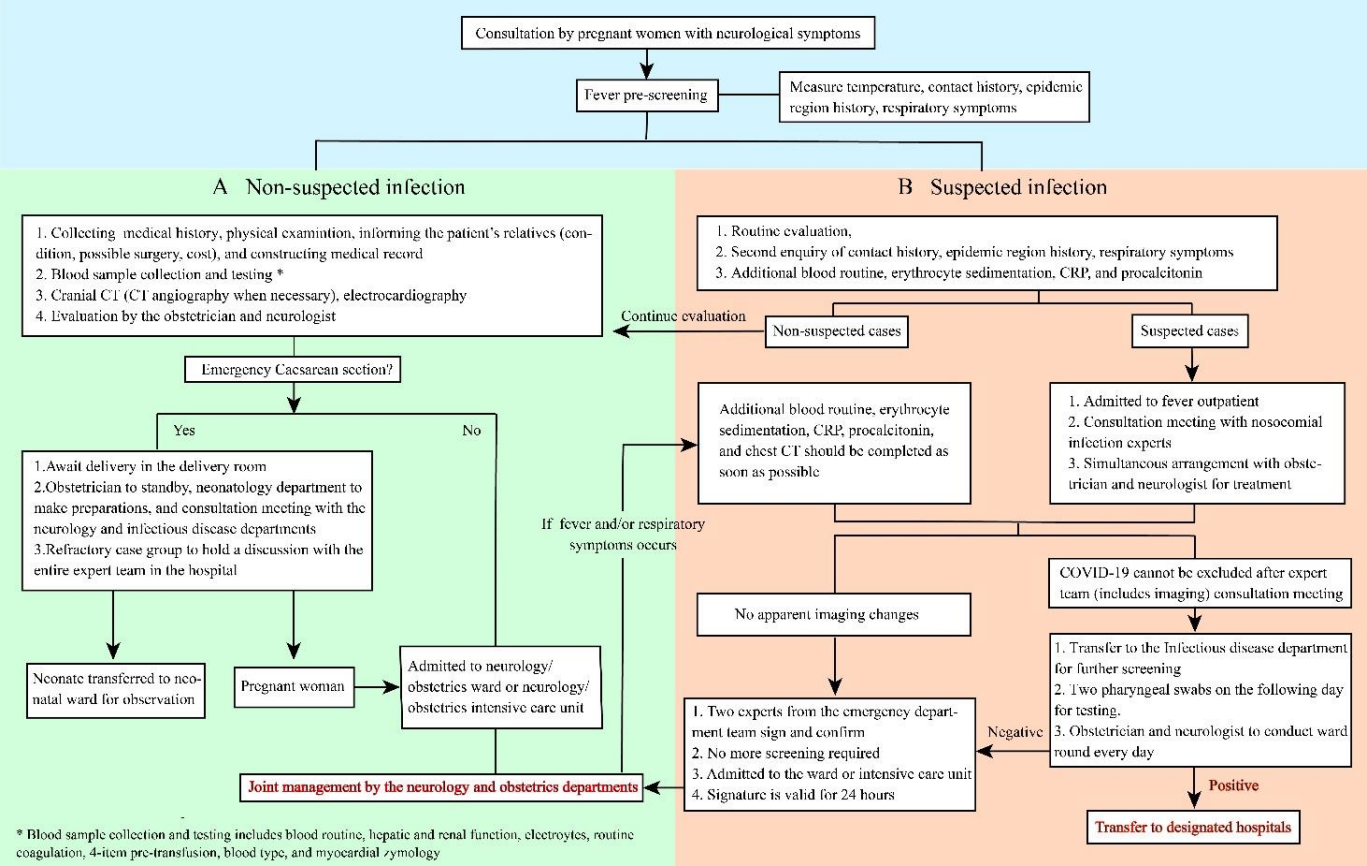
Admission and treatment flowchart for pregnant women with complicated neurological symptoms.

### 5.1 Strengthen personnel flow control and standardize admission criteria

As the neurological symptoms of pregnant women with complicated neuropathy are similar to those CNS complaints observed in COVID-19-infected pregnant women, the identification and triage of those patients should be strengthened and started during admission. We propose a flowchart in Figure 1 to show the admission and treatment process.

### 5.2 Strengthen inpatient management

At present, though COVID-19 vaccines show some protective effects, new SARS-CoV-2 variants with high fatality and high risk of infection, such as Delta and Omicron, still threat the global health [83, 84]. Therefore, strict prevention and control systems still need to be implemented. If a pregnant patient is suspected with COVID-19 infection, she should be quarantined in a specially designed isolation ward immediately. The activities of the patient and family members should be restricted. Medical staff should wear protective gears (N95 mask and disposable isolation gown as primary protection) and avoid repeated entry of the ward. To confirm the diagnosis and evaluate her condition, emergency chest MRI and blood routine, ordinary CRP, erythrocyte sedimentation, procalcitonin, 4-item pre-transfusion, and electrolyte tests should be performed. Fetal heart monitoring, Four-Step palpation, abdominal ultrasound, and necessary obstetric examinations should be taken to evaluate the condition of the foetus. Following points should be emphasized: (1) patients should be quarantined immediately, (2) inpatients should be guided on correctly selecting and wearing masks, cough etiquette, and hand hygiene, (3) non-necessary visit and personnel control should be banned, (4) epidemic control knowledge should be proactively advocated and (5) multidisciplinary cooperation should be considered for refractory cases.

## 6. Popularising online diagnosis and treatment

To reduce the risk of transmission, hospitals have established online consultation systems. The ‘Healthy China 2020’ plan pointed out that prevention should be the key focus and encouraged to improve the public’ health awareness [85]. Midwifery institutions can establish online obstetric clinics and remote consultations. Through joint consultations with neurology, obstetrics, and neonatology, they can provide effective and sufficient health education for pregnant women to ensure their safety during the epidemic.

## 7. Conclusion

Pregnant women have altered immune functions are susceptible to various pathogens. Besides, they are among the groups with low vaccination rates, making them a COVID-19-susceptible population. The incidence of neurological symptoms in pregnant women without previously diagnosed or newly diagnosed neurological disease is about 2% [86]. Those symptoms are much like neurological symptoms caused by the neuro-invasive virus, SARS-CoV-2. Treatments applied in these two situations are quite different. Therefore, distinguishing pregnant women with neuropathy and neurological symptoms caused by COVID-19 is rather important as well as a challenge for obstetricians. At present, no specific prophylactic drug for managing SARS-CoV-2 is available. Therefore, strengthening the identification of patients with COVID-19 during clinical practice is critical in preventing nosocomial and cross-infections in pregnant women. The emergency management framework we proposed in this article aims at improving the efficacy of emergency guarantee system under COVID-19 pandemic and protecting the health of both the pregnant patients as well as the medical staff. This system could also serve as a potential multisystem project for clinical practice and medical education.
